# Phylogenetic Analysis of the Mpox Virus in Sub-Saharan Africa (2022–2024)

**DOI:** 10.3390/biology14070773

**Published:** 2025-06-26

**Authors:** Millicent Ochieng, Daniel Kiboi, Carolyne Nasimiyu, Eric Osoro, Dorcus C. A. Omoga, Josiah O. Kuja

**Affiliations:** 1 Paul G. Allen School for Global Health–Kenya, College of Veterinary Medicine, Washington State University, Nairobi 00200, Kenya; 2 Department of Biochemistry, School of Biomedical Sciences, Jomo Kenyatta University of Agriculture and Technology, P.O. Box 62000–00200, Nairobi 00200, Kenya; 3 Directorate of Veterinary Services, Nairobi 00100, Kenya

**Keywords:** Mpox virus, MPXV evolution, genetic diversity, APOBEC3 mutations, immune evasion, phylogenetics, protein stability

## Abstract

Mpox, caused by the Mpox virus (MPXV), is a re-emerging zoonotic disease in the Poxviridae family. Since 2022, sub-Saharan Africa has experienced recurrent outbreaks, with the Democratic Republic of the Congo (DRC) accounting for 96% of the 567 confirmed cases reported in the African region by the World Health Organization as of June 2024. Despite MPXV’s endemic presence, its genomic diversity and evolutionary dynamics remain poorly characterized. We analyzed 270 MPXV genomes from 13 sub-Saharan African countries (2022–2024), representing the most geographically comprehensive regional dataset from the outbreak period. Phylogenetic analysis identified two geographically distinct clades: Clade I (East/Central Africa) and Clade II (West/Southern Africa). A marked disparity in APOBEC3-associated mutations was observed, with Clade IIb exhibiting significantly higher enrichment than Clade I, suggesting clade-specific host adaptation pressures. These mutations predominantly target genes involved in immune evasion and replication. Preliminary functional predictions indicated that selected missense mutations may impact on protein stability, underscoring the need for further experimental validation. Our findings provide the first pan–sub-Saharan analysis of MPXV clade divergence and reinforce the importance of sustained, regionally informed genomic surveillance to monitor viral evolution and guide outbreak response strategies across Africa.

## Simple Summary

Mpox is a virus that has caused repeated outbreaks in parts of Africa, especially in the Democratic Republic of Congo. Despite being known for some time, how the virus changes and adapts is not well understood. In this study, we examined the genetic information of Mpox virus from 13 African countries to see how they differ and evolve. We identified two major types of the virus, with one detected in East and Central Africa and the other in West and Southern Africa. We also discovered changes in the virus’ genes that may help it avoid the immune system or affect how it replicates. These insights can help improve the monitoring of Mpox outbreaks and support better public health responses. However, more laboratory work is needed to confirm the effects of these genetic changes.

## 1. Introduction

MPXV is a double-stranded DNA virus of the family Poxviridae, subfamily Chordopoxvirinae, and genus Orthopoxvirus, which also includes Variola virus (smallpox), Vaccinia virus (used in smallpox vaccines), and Cowpox virus [1]. First identified in 1958 in captive monkeys in Copenhagen, MPXV became a recognized human pathogen in 1970, following an infection in a 9-month-old child in the DRC [2]. Historically, mpox infections were restricted to Central and West Africa, typically resulting from zoonotic spillovers with limited human-to-human transmission. However, in 2022, a significant epidemiological shift occurred. Mpox cases surged globally, prompting the World Health Organization (WHO) to declare it a Public Health Emergency of International Concern (PHEIC) [3,4].

MPXV is a large, linear double-stranded DNA virus with a ~190 kb genome encoding over 190 proteins (Figure S1). Its genome has a conserved central region (~32–138 kb) that encodes essential replication enzymes and structural proteins, flanked by variable terminal regions containing inverted terminal repeats (ITRs) involved in host interaction, immune evasion, and virulence [1,5] (Figure S2). Open reading frames (ORFs) follow the standard orthopoxvirus nomenclature (e.g., OPG001) [6]. Key ORFs encode DNA and RNA polymerases, envelope proteins, and virulence factors such as complement regulatory proteins [1,7] (File S1).

Mpox is classified into two principal clades designated as Clade I (Central African) and Clade II (West African), with further subdivisions into Clades Ia, Ib, IIa, and IIb, respectively [8]. Clade I is historically associated with higher morbidity and mortality, disproportionately affecting children and immunocompromised individuals, whereas Clade II has been implicated in the recent global outbreaks, particularly among men who have sex with men (MSM) [8]. Mpox however remains endemic in the sub-Saharan countries including DRC, Central African Republic, Cameroon, and Nigeria, with reported cases in Ghana [9]. The natural reservoir of MPXV remains unknown, although zoonotic transmission is suspected to be caused by African rodents and non-human primates [10].

On a global scale, multiple mpox outbreaks have been reported in over 130 countries by April 2025, with sustained human-to-human transmission [11]. The 2022–2024 outbreaks in Europe, North America, and Asia also demonstrated novel transmission dynamics, including sexual transmission networks and asymptomatic carriers [11,12,13]. While Clade II remains dominant in these outbreaks, more recent genomic analyses have identified mutations consistent with APOBEC3 cytidine deaminase activity, characterized by GA-to-AA and TC-to-TT substitutions [1].

Several public health efforts have been coordinated by WHO to curb the spread of mpox. These include targeted vaccination strategies, enhanced surveillance, and support for rapid diagnostic capacities. However, vaccine access remains inequitable, particularly in endemic regions of Africa [14,15]. By August 2024, over 20,000 suspected cases and 500 deaths had been reported in the DRC [16], hence emphasizing the important need for increased genomic surveillance, equitable vaccine distribution, and international collaboration to mitigate mpox outbreaks.

Despite recent advances, the genetic evolution and diversity of MPXV across sub-Saharan Africa remain underexplored. Most phylogenomic studies have focused on Clade IIb viruses from non-endemic countries [17,18,19,20], while African studies have largely been limited to single-country datasets [21,22,23,24], restricting broader evolutionary insights. To address this gap, we conducted the first pan–sub-Saharan genomic analysis of 270 MPXV genomes from 13 countries (2022–2024). This study investigates regional lineage divergence, APOBEC3-mediated mutational signatures, and the potential functional effects of key non-synonymous mutations. Our findings provide critical insights into MPXV’s evolutionary dynamics and underscore the importance of genomic surveillance, vaccine updates, and outbreak preparedness across both endemic and emerging regions.

## 2. Materials and Methods

### 2.1. Phylogenetic Analysis

Complete genome sequences of the MPXV were retrieved from GISAID EpiPox (400 sequences) (Global Initiative on Sharing All Influenza Data) (https://gisaid.org/) (accessed on 4 March 2025) covering the period between 1 January 2022, and 31 December 2024. Accession numbers were selected from 13 African countries with high Mpox prevalence throughout this time interval. Sequences underwent several curation processes for data quality. Duplicate sequences were eliminated. Sequences of sizes ranging from 190 to 205 kb were kept ensuring complete and consistent genomes. Sequences containing more than 10% ambiguous nucleotides (Ns) were excluded to ensure high-confidence data. Sequence preprocessing was carried out using Biopython scripts (Python 3.9) [25]. After curation, 270 sequences were kept for further analysis including: DRC (*n* = 167), Nigeria (*n* = 38), Central African Republic (*n* = 14), Burundi (*n* = 9), South Africa (*n* = 9), Ghana (*n* = 7), Uganda (*n* = 7), Cote d’Ivoire (*n* = 5), Cameroon (*n* = 4), Liberia (*n* = 5), Benin (*n* = 2), Kenya (*n* = 2), and Congo (*n* = 1).

Multiple sequence alignment (MSA) was performed with MAFFT v7.526 [26] using the auto strategy to optimize speed with an assurance of accuracy. TrimAl v1.4. rev15 (build 17 December 2013) [27] was used to filter the alignment, applying a 0.05 gap threshold to remove poorly aligned regions. The quality of the alignment was visually inspected, using AliView v1.26 to ensure accuracy prior to downstream analyses [28].

A maximum likelihood (ML) tree was constructed using IQ-TREE v2.3.6 [29]. The best-fitting nucleotide substitution model K3Pu+F+I+R3 was selected using ModelFinder [30] with Bayesian Information Criterion (BIC) values for the best fit to the data. For tree robustness, 1000 ultrafast bootstrap replicates were performed [31]. The 2016 sequence (EPI_ISL_18689512) was used as an outgroup to root the phylogenetic tree and for evolutionary context. The phylogenetic tree was visualized and annotated using iTOL v7.1 with metadata [32].

### 2.2. Mutational Analysis

Mutational analysis was performed by aligning the reference sequence NC_063383.1 (MPXV, 2018) with 12 representative mutant sequences for each lineage and clades using BWA for indexing and alignment v0.7.17-r1188 [33]. Representative sequences were randomly selected to maintain manageable dataset subsets for mutational analysis. The aligned sequences were converted from SAM to BAM format, sorted, and indexed. Variant calling was carried out with BCF tools v1.19 [34], followed by variant annotation using snpEff v5.2e [35]. A custom Mpox sequence database was compiled to support variant annotation, using the standard genetic code. A Python script was developed to rapidly screen for APOBEC3 mutation signatures across lineages and clades, available in the Supplementary Material (File S3) and at our GitHub repository: https://github.com/Millicent-lab/mpox-apobec3-screening (version a1ab764, accessed on 23 May 2025).

### 2.3. Functional Analysi

To assess the potential biological impact of non-synonymous amino acid substitutions identified across MPXV genes, we conducted a preliminary in silico functional analysis. From the mutational dataset, six genes with the highest number of amino acid substitutions were prioritized. For each gene, three consensus mutations were selected based on their recurrence across multiple clades and lineages (File S2). Functional predictions for each amino acid substitution were obtained using PolyPhen-2 (http://genetics.bwh.harvard.edu/pph2/, accessed on 21 May 2025). The mutations were classified as “benign”, “possibly damaging”, or “probably damaging” based on the associated score [36].

Two viral genes were selected for structural modeling based on the high frequency of missense mutations and the presence of at least two variants predicted to be damaging by PolyPhen-2. Due to the lack of suitable homologous templates in the Protein Data Bank (PDB), protein structure predictions were generated using a publicly available AlphaFold-based web server (https://alphafoldserver.com/, accessed on 29 May 2025), which utilizes the AlphaFold2 [37] algorithm for tertiary structure prediction, using the MPXV reference genome NC_063383.1 as input. Model quality was evaluated using pLDDT scores [37], where values above 70 generally indicate reliable backbone predictions, and further validated through stereochemical assessment using PROCHECK [38].

The impact of the mutations on protein stability was assessed using the DynaMut web server (https://biosig.lab.uq.edu.au/dynamut2, accessed on 27 May 2025) [39]. Wild-type structures were used as input, and predicted changes in Gibbs free energy (ΔΔG) were calculated to estimate the impact of mutations on protein stability. Positive ΔΔG values indicate stabilizing mutations, while negative values indicate destabilizing effects.

Conservation scores were calculated for the wild-type protein to provide a comprehensive view of residue conservation using the ConSurf web server (https://consurf.tau.ac.il, accessed on 27 May 2025) [40] based on the MSA of orthologous Orthopoxvirus proteins.

## 3. Results

### 3.1. Phylogenetic Analysis

**Figure 1:**
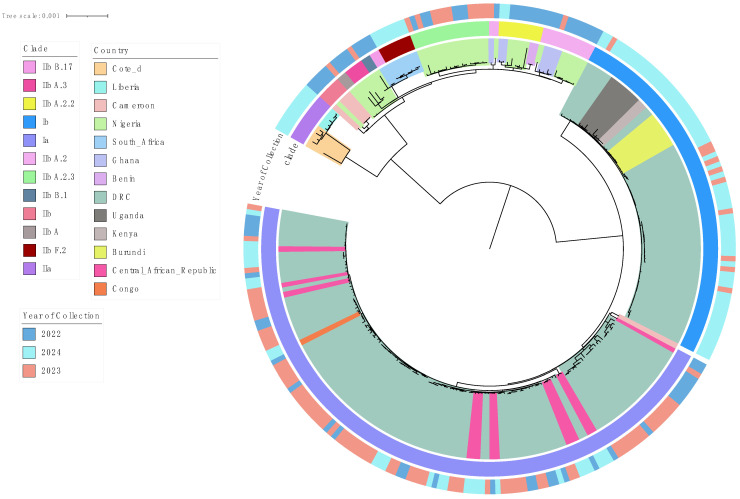
Phylogenetic tree of MPXV sequences (January 2022–December 2024) retrieved from GISAID on 4 March 2025. A long divergent branch marks the transition from Lineage A.3 (pink) to B.1 (gray blue). Inner most shadings indicate the country of collection.

**Figure 2:**
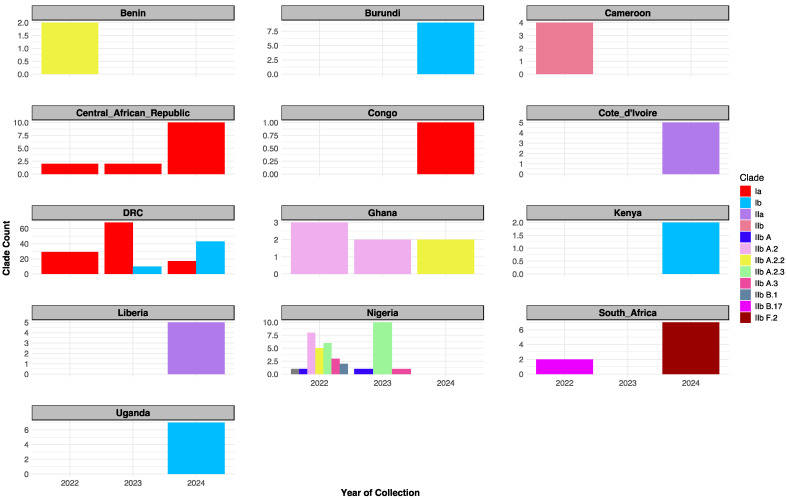
Temporal distribution of MPXV clades in Sub-Saharan Africa (2022–2024). Clade IIb dominated in 2022 (notably in Nigeria); Clade Ia peaked in 2023 then declined; Clade Ib rose in 2023 and peaked in 2024; Clade I remained prevalent in DRC through 2022 to 2024.

Phylogenetic analysis of 270 curated MPXV sequences from Sub-Saharan Africa identified two major clades: Clade I (Ia, Ib) and Clade II (IIa, IIb), with distinct evolutionary and temporal patterns (Figure 1 and Figure 2). Notably a long divergent branch marked the transition from Lineage A.3 to B.1. Clade I (Ia and Ib) was predominant in Central and East African countries such as the Democratic Republic of the Congo (DRC), Uganda, Burundi, Central African Republic (CAR), Kenya, and the Republic of Congo. Clade II was mainly observed in West and Southern Africa, with IIb reported in countries including South Africa, Nigeria, Ghana, Benin, and Cameroon, and IIa detected in Liberia and Côte d’Ivoire.

Clade IIb diverged into several sub-lineages; A, A.2, A.2.2, A.2.3, A.3, B.1, B.17, and F.2 as defined by the GISAID EpiPox classification and supported by strong phylogenetic clustering and high bootstrap values (https://gisaid.org/hmpxv-epipox, accessed on 27 May 2025) [23] (File S4).

Temporal distribution analysis revealed evolving lineage dynamics across the three-year period (Figure 2). In 2022, multiple lineages co-circulated, including Clade Ia, IIb, and several IIb sub-lineages (A, A.2, A.2.2, A.2.3, A.3, B.1, and B.17). In 2023, Clade Ia peaked, Clade Ib emerged, and IIb sub-lineages A, A.2, A.2.3, and A.3 remained in circulation. By 2024, Clade Ia declined while Clade Ib became dominant. Notably, Clade IIa appeared exclusively in 2024, alongside the continued circulation of IIb sub-lineages A.2.2 and F.2 (Table S1).

### 3.2. Mutational Analysis

**Figure 3:**
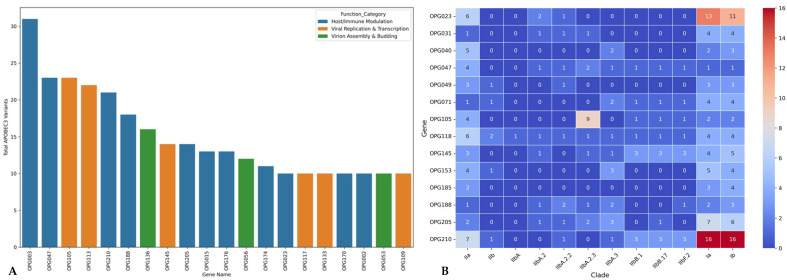
(**A**) Bar plot shows the top MPXV genes based on the frequency of APOBEC3-associated mutations. These mutations, known to arise from host antiviral activity, were predominantly observed in genes involved in immune modulation (blue) and viral replication/transcription (orange). (**B**) Heatmap illustrates the cumulative frequency of non-synonymous (missense) mutations across major MPXV genes and clades, using NC_063383.1 (Clade IIbA) as the reference genome. The color intensity corresponds to the absolute number of missense mutations per gene per clade, with darker shades indicating higher mutation counts. Notably, OPG210 showed the highest mutation frequency overall, particularly within Clade I, followed by OPG023 and OPG105 which showed pronounced mutation levels in Clade I and Clade IIbA.2.3, respectively.

The mutational analysis of MPXV genomes revealed notable non-synonymous amino acid substitutions relative to the reference genome NC_063383.1 (MPXV, 2018). A focused examination of consensus mutations identified the most frequently altered genes across clades, selected based on their cumulative missense mutation burden. The OPG210 gene exhibited the highest mutation frequency, followed by OPG023, OPG118, OPG145, OPG205, OPG105, OPG153, OPG071, OPG047, and OPG188 (Figure 3B).

Clade-specific mutational patterns emerged, with Clade I showing the highest number of substitutions overall, particularly in OPG023, OPG210, and OPG205. Within Clade IIb, Lineage B.1 displayed elevated mutation counts in OPG210 compared to Lineage A, while Lineage IIbA.2.3 exhibited the greatest number of substitutions in OPG105 relative to the reference genome (Figure 3B).

Overall, 54.65% of observed mutations were synonymous, while 45.35% were non-synonymous. Among these, 34.55% of synonymous and 41.89% of non-synonymous mutations bore APOBEC3-associated mutation signatures (Table 1). Clade IIb harbored the highest proportion of APOBEC3-mediated variants, particularly in Lineage B.1 and its sub-lineages (B.17 and F.2), which averaged 88.34% of synonymous and 90.14% of non-synonymous mutations. Conversely, Clade I exhibited the lowest proportions of APOBEC3-associated variants, with averages of 27.54% (synonymous) and 25.69% (non-synonymous). Genes enriched for APOBEC3 mutations were predominantly those involved in host immune modulation as well as viral replication and transcription (Figure 3A).

### 3.3. Functional Analysis

**Figure 4:**
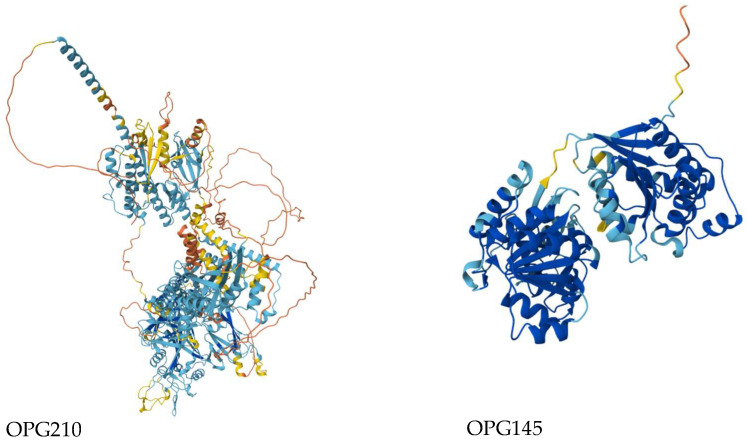
Predicted 3D wildtype structures of OPG210 (left) and OPG145 (right) colored by pLDDT confidence scores. Dark blue: very high confidence (pLDDT > 90); light blue: confident (70–90); yellow: low confidence (50–70); orange: very low confidence (<50).

While the majority of substitutions were predicted to be benign, several exhibited high PolyPhen-2 scores (File S8), suggesting potential functional impacts. These included, R689C in OPG205 (0.999), L602I in OPG023 (0.659), S734L in OPG105 (0.683), R243Q (0.996) and E435K (0.948) in OPG145, and D209N (0.999) and P722S (0.640) in OPG210 (Files S2 and S6) [28]. OPG210 and OPG145 were prioritized for structural and stability analyses as both harbored two high-impact mutations and are functionally implicated in immune modulation and DNA helicase activity, respectively. Protein structure predictions yielded overall pLDDT scores of 65.94 for OPG210 and 86.41 for OPG145, corresponding to low and high-confidence models, respectively (Figure 4; File S6) [29]. While local stereochemical features of both models were acceptable (File S7) [30], the global confidence of the OPG210 structure was limited. Stability predictions were subsequently conducted to evaluate the structural impact of these variants, although results for OPG210 should be interpreted with caution due to its model’s lower reliability.

**Figure 5:**
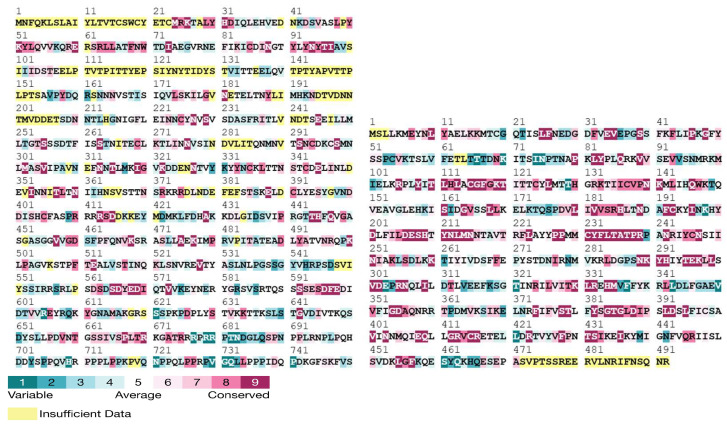
Conservation profiles of OPG210 (1–750 aa segment shown) and full-length OPG145 (1–492 aa) generated using the ConSurf web server. Conserved residues are color coded based on evolutionary conservation scores.

Stability and conservation analyses indicated that the D209N mutation in OPG210 was stabilizing (+1.2 kcal/mol) and located at a mildly variable site adjacent to a highly conserved region. Conversely, the P722S substitution in OPG210 was slightly destabilizing (−0.16 kcal/mol) and occurred within a highly variable region. In OPG145, the R243Q mutation was destabilizing (−0.54 kcal/mol) and mapped to a highly conserved residue, whereas E435K was stabilizing (+0.51 kcal/mol) and situated within a moderately conserved region flanked by conserved residues (Figure 5; File S6) [30,31].

## 4. Discussion

Our pan–sub-Saharan phylogenetic analysis confirms the co-circulation of Clade I (Ia/Ib) and Clade II (IIa/IIb) and offers new insights into their regional distribution and evolutionary trajectories. While prior studies have described MPXV genetic diversity [1,17,18,19,20], this is the first to resolve lineage-specific patterns across 13 endemic African countries. We corroborate Isidro et al.’s [18] (p. 1) observation of a long divergent branch separating Lineage A from B.1, indicative of accelerated microevolution (Figure 2). We extend this finding by identifying a higher burden of non-synonymous substitutions in the immune evasion gene OPG210 in Lineage B.1 compared to Lineage A (Figure 3B; Figure S2). This suggests ongoing adaptive evolution potentially shaped by regional host immune pressures, an underexplored pattern in African MPXV strains.

While Clade I’s endemicity in Central Africa and the DRC is well established [14], our analysis reveals broader regional clustering of Clade Ib strains across Kenya, Burundi, Uganda, and the DRC (Figure 2) [41]. Although previous in-country reports have noted possible cross-border transmission [42,43,44], this study provides the most geographically expansive phylogenetic evidence of Clade Ib circulation across East and Central Africa. However, the absence of linked metadata such as travel history or case contact information limits definitive conclusions about transmission routes. These findings underscore the need for integrated genomic and epidemiological surveillance to better understand MPXV spread dynamics in sub-Saharan Africa.

Our analysis confirms the predominance of APOBEC3-mediated mutations (GA>AA/TC>TT) in Clade IIb Lineage B.1 [1,18], and reveals gene-specific APOBEC3 editing, with selective enrichment in immune-modulatory genes OPG210, OPG047, and OPG003 (Figure 3A). Clade IIb exhibits approximately a threefold higher mutation burden than Clade I (Table 1), supporting the hypothesis of host-driven adaptation [45,46], particularly in viral immune evasion proteins. Functional validation of these mutations’ fitness impacts remains essential. Conversely, Clade I shows a low APOBEC3 editing burden (<30%), dominated instead by non-canonical mutations (File S5), indicating alternative mutational strategies. Similar phenomena have been observed in gamma-herpesviruses [47] and HIV-1 [48] under comparable selective pressures. The discordance between Clade I’s low APOBEC3 signature and its expanding geographic range implies alternative adaptation mechanisms such as enhanced polymerase fidelity or reservoir-specific selection that warrant systematic investigation.

We also observed that MPXV evolution is shaped by differential selection on synonymous (54.65%) and non-synonymous (45.35%) mutations (Table 1). Although synonymous mutations do not alter protein sequences, they may impact viral fitness through codon usage bias, RNA structural stability, or translational efficiency [49,50]. This is supported by their distinct distribution in virulent Central African versus attenuated West African strains [51]. Notably, non-synonymous mutations predominate in Clade IIb B.1 isolates relative to the 2018 reference genome (NC_063383.1) (Figure 3B), suggesting strong positive selection driving protein evolution. This trend aligns with recent findings of accelerated amino acid substitutions and reduced codon adaptation index (CAI) in 2022 outbreak strains [52,53], implying that MPXV may be undergoing an adaptive shift in which protein innovation supersedes codon optimization during host adaptation. Together, these findings highlight the interplay of mutation types in shaping MPXV evolution and pathogenicity.

Clade-specific mutations further illuminate potential adaptive pathways in MPXV. Compared to the 2018 Clade IIb reference (NC_063383.1), recent Clade IIb A.2.3 strains harbor concentrated mutations in OPG105 (Figure 3B), a transcriptional regulator (Figure S2) potentially involved in replication optimization [20,54]. Meanwhile, Clade I (hyperendemic in Africa) exhibits distinct variation in immune-related genes OPG210, OPG205, and OPG023 (File S2; Figure 3B), reflecting both ancestral divergence and recent adaptation. Similar gene changes have been shown to enhance viral fitness in other poxviruses [55]. These genetic signatures may underpin clade-specific transmission patterns, although functional validation remains necessary.

Several methodological limitations should be acknowledged. Our reliance on a single reference genome and consensus sequences limited the detection of lineage-specific and low-frequency variants, potentially obscuring intra-host diversity. The absence of raw sequencing data further restricted variant resolution. Additionally, APOBEC3 mutation profiling was hindered by incomplete metadata, precluding correlation with clinical or epidemiological outcomes. Nevertheless, the mutational hotspots identified here represent candidates of potential functional significance. Future studies incorporating deep sequencing and richer patient-level data will be vital to fully elucidate MPXV evolutionary dynamics.

Our integrated functional analysis framework offers mechanistic insights into MPXV protein evolution. We identify the R243Q mutation in OPG145, predicted to be destabilizing (ΔΔG = –0.54 kcal/mol) and occurring at a universally conserved residue (Figure 5) that is indicative of strong evolutionary constraint. This mirrors replication-defective D5 helicase mutants in vaccinia virus, such as Dts6389, where insolubility and impaired multimerization disrupt replication [56]. Given OPG145’s confirmed helicase function (Figure S2), the R243Q mutation likely impairs viral replication through similar biophysical mechanisms, a hypothesis testable via ATPase activity and thermal shift assays.

We also identify stabilizing mutations, E435K in OPG145 (ΔΔG = +0.51 kcal/mol) and D209N in OPG210 (ΔΔG = +1.2 kcal/mol) located near conserved functional regions (Figure 5). These may counterbalance destabilizing variants, exemplifying compensatory stabilization [57,58,59] (File S6). In contrast, mildly destabilizing variants such as OPG210-P722S (ΔΔG = –0.16 kcal/mol) located in highly variable regions, though individually tolerated, may cumulatively shape protein evolution. As noted by Tokuriki and Tawfik [59] (p. 1), mutations that cross a destabilization threshold can reduce protein solubility and impair its function. Although the structural resolution of the OPG210 model limits atomic interpretation, our integrative approach highlights OPG145-R243Q and OPG210-D209N as priority targets for cryo-EM structural studies and functional assays. These findings offer a methodological framework for dissecting MPXV protein function through integrated structural and evolutionary approaches.

## 5. Conclusions

This study underscores the ongoing evolution of MPXV in sub-Saharan Africa, characterized by the diversification of Clade IIb and the emergence of region-specific sub-lineages. These trends reflect the virus’ adaptability across diverse ecological and geographic landscapes. The predominance of APOBEC3-mediated mutations and the elevated burden of non-synonymous substitutions suggest sustained host-driven adaptation, likely influenced by immune selection pressures and evolving transmission dynamics.

These genomic signatures serve as early indicators of potential shifts in viral behavior and carry direct public health significance. They highlight the urgency of real-time genomic surveillance to detect emerging variants, inform vaccine updates, and enhance outbreak preparedness, particularly in endemic and high-risk regions.

Importantly, our findings raise critical questions about the functional consequences of mutations in immune-modulatory and transcriptional regulatory genes. Future work should prioritize experimental validation of these candidates to determine their roles in MPXV transmissibility and pathogenicity. Continued integration of genomic and epidemiological data will be essential to inform targeted and timely public health responses.

## Figures and Tables

**Table 1:** Distribution of synonymous, non-synonymous, and APOBEC3 variants across lineages/clades.

Clade/Lineage	Synonymous Variants	% APOBEC3 (Synonymous)	Non-Synonymous Variants	% APOBEC3 (NON-Synonymous)
IIa	137	29.20% (40/137)	124	25.81% (32/124)
IIbA.2.3	15	73.33% (11/15)	31	74.19% (23/31)
IIbA.2	12	75.00% (9/12)	22	95.45% (21/22)
IIbA.3	28	46.43% (13/28)	55	38.18% (21/55)
IIbA	7	0.00% (0/7)	8	25.00% (2/8)
IIb	23	13.04% (3/23)	23	8.70% (2/23)
Ia	364	26.65% (97/364)	240	24.17% (58/240)
IIbB1	27	88.89% (24/27)	32	90.63% (29/32)
IIbF.2	38	86.84% (33/38)	33	90.91% (30/33)
IIbB.17	28	89.29% (25/28)	36	88.89% (32/36)
Ib	380	28.42% (108/380)	261	27.20% (71/261)
IIbA.2.2	9	66.67% (6/9)	21	95.24% (20/21)
Total	1068(54.65%)	34.55% (369/1068)	886(45.35%)	41.89% (371/886)

## Data Availability

The data presented in this study is available in the Supplementary Materials.

## Supplementary Materials

The following supporting information can be downloaded at: https://www.mdpi.com/article/10.3390/biology14070773/s1. File S1: Supplementary Figures (zip file containing detailed supplementary figures for the schematic diagram of NC_063383.1). Figure S1: Schematic representation of the MPXV genome architecture, indicating ORF, CDS, and miscellaneous regions, generated using Proksee; Figure S2: Genomic functionality, color-coded based on functionality, generated using Python’s Matplotlib. File S2: Mutational analysis files (zip file containing detailed analysis). File S3: APOBEC3 filter, Python script used for variant filtering. File S4: Phylogenetic analysis files (zip file containing the phylogenetic analysis data). File S5: APOBEC3 variants data (zip file with variants). Table S1: Metadata used for sequence analysis (Excel file). File S6: Functional analysis files (Zip file containing all the functional analyses files). File S7: Ramachandran plot of the predicted 3D structure of OPG 210 and OPG 145 as evaluated by PROCHECK. File S8: PolyPhen-2 scores for selected non-synonymous mutations in highly mutated genes.

## Abbreviations

The following abbreviations are used in this manuscript:

Abbreviation	Full Term
MPXV	Monkeypox Virus
APOBEC3	Apolipoprotein B mRNA Editing Enzyme Catalytic Subunit 3
OPG	Orthopoxvirus Gene
DRC	Democratic Republic of the Congo
WHO	World Health Organization
BIC	Bayesian Information Criterion
CAR	Central African Republic
ML	Maximum Likelihood
MSA	Multiple Sequence Alignment
MSM	Men Who Have Sex with Men
PHEIC	Public Health Emergency of International Concern
ITR	Inverted terminal repeats
pLDDT	predicted Local Distance Difference Test
MAFFT	Multiple Alignment using Fast Fourier Transform
iTOL	Interactive Tree of Life
PROCHECK	Protein Checking program
Cryo-EM	Cryogenic Electron Microscopy

## References

[1] Alakunle E., Kolawole D., Diaz-Cánova D., Alele F., Adegboye O., Moens U., Okeke M.I. (2024). A comprehensive review of monkeypox virus and mpox characteristics. Front. Cell. Infect. Microbiol..

[2] Martínez-Fernández D.E., Fernández-Quezada D., Casillas-Muñoz F.A.G., Carrillo-Ballesteros F.J., Ortega-Prieto A.M., Jimenez-Guardeño J.M., Regla-Nava J.A. (2023). Human Monkeypox: A Comprehensive Overview of Epidemiology, Pathogenesis, Diagnosis, Treatment, and Prevention Strategies. Pathogens.

[3] Jadhav V., Paul A., Trivedi V., Bhatnagar R., Bhalsinge R., Jadhav S.V. (2025). Global epidemiology, viral evolution, and public health responses: A systematic review on Mpox (1958–2024). J. Glob. Health.

[4] World Health Organization WHO Director-General Declares Mpox Outbreak a Public Health Emergency of International Concern. WHO 2024, August 14. https://www.who.int/news/item/14-08-2024-who-director-general-declares-mpox-outbreak-a-public-health-emergency-of-international-concern.

[5] Shchelkunov S.N., Totmenin A.V., Safronov P.F., Mikheev M.V., Gutorov V.V., Ryazankina O.I., Petrov N.A., Babkin I.V., Uvarova E.A., Sandakhchiev L.S. (2002). Analysis of the monkeypox virus genome. Virology.

[6] Happi C., Adetifa I., Mbala P., Njouom R., Nakoune E., Happi A., Ndodo N., Ayansola O., Mboowa G., Bedford T. (2022). Urgent need for a non-discriminatory and non-stigmatizing nomenclature for monkeypox virus. PLoS Biol..

[7] Senkevich T.G., Yutin N., Wolf Y.I., Koonin E.V., Moss B. (2021). Ancient Gene Capture and Recent Gene Loss Shape the Evolution of Orthopoxvirus-Host Interaction Genes. mBio.

[8] Duarte P.M., Adesola R.O., Priyadarsini S., Singh R., Shaheen M.N.F., Ogundijo O.A., Gulumbe B.H., Lounis M., Samir M., Govindan K. (2024). Unveiling the Global Surge of Mpox (Monkeypox): A comprehensive review of current evidence. Microbe.

[9] Africa CDC Outbreak Report: Mpox SSituation in Africa. Africa CDC 2024, July 30. https://africacdc.org/disease-outbreak/mpox-situation-in-africa/.

[10] Khodakevich L., Szczeniowski M., Jezek Z., Marennikova S., Nakano J., Messinger D. (1987). The role of squirrels in sustaining monkeypox virus transmission. Trop. Geogr. Med..

[11] World Health Organization (1 April 2025). Global Mpox Trends. https://worldhealthorg.shinyapps.io/mpx_global/#8_Disclaimers.

[12] Satapathy P., Mohanty P., Manna S., Shamim M.A., Rao P.P., Aggarwal A.K., Khubchandani J., Mohanty A., Nowrouzi-Kia B., Chattu V.K. (2022). Potentially Asymptomatic Infection of Monkeypox Virus: A Systematic Review and Meta-Analysis. Vaccines.

[13] Vaughan A.M., Afzal M., Nannapaneni P., Leroy M., Andrianou X., Pires J., Funke S., Roman C., Reyes-Uruena J., Aberle S. (2024). Continued Circulation of Mpox: An Epidemiological and Phylogenetic Assessment, European Region, 2023 to 2024. Eurosurveillance.

[14] Bunge E.M., Hoet B., Chen L., Lienert F., Weidenthaler H., Baer L.R., Steffen R. (2022). The Changing Epidemiology of Human Monkeypox—A Potential Threat? A Systematic Review. PLOS Neglected Trop. Dis..

[15] Olawade D.B., Wada O.Z., Fidelis S.C., Oluwole O.S., Alisi C.S., Orimabuyaku N.F., Clement David-Olawade A. (2024). Strengthening Africa’s Response to Mpox (Monkeypox): Insights from Historical Outbreaks and the Present Global Spread. Sci. One Health.

[16] Tiwari A., Kalonji T., Miller T., Van Den Bossche T., Krolicka A., Muhindo-Mavoko H., Mitashi P., Tahita M.C., Lood R., Pitkänen T. (2025). Emergence and Global Spread of Mpox Clade Ib: Challenges and the Role of Wastewater and Environmental Surveillance. J. Infect. Dis..

[17] Gigante C.M., Korber B., Seabolt M.H., Wilkins K., Davidson W., Rao A.K., Zhao H., Smith T.G., Hughes C.M., Minhaj F. (2022). Multiple lineages of monkeypox virus detected in the United States, 2021–2022. Science.

[18] Isidro J., Borges V., Pinto M., Sobral D., Santos J.D., Nunes A., Mixão V., Ferreira R., Santos D., Duarte S. (2022). Phylogenomic characterization and signs of microevolution in the 2022 multi-country outbreak of monkeypox virus. Nat. Med..

[19] Luna Niño N., Ramírez A., Muñoz M., Ballesteros N., Patiño L., Castañeda Garzon S., Bonilla-Aldana D., Paniz-Mondolfi A., Ramírez J. (2022). Phylogenomic analysis of the monkeypox virus (MPXV) 2022 outbreak: Emergence of a novel viral lineage?. Travel Med. Infect. Dis..

[20] Wang L., Shang J., Weng S., Aliyari S.R., Ji C., Cheng G., Wu A. (2023). Genomic annotation and molecular evolution of monkeypox virus outbreak in 2022. J. Med. Virol..

[21] Chan W.Y., Mtshali P.S., Grobbelaar A., Moolla N., Mohale T., Lowe M., Du Plessis M., Ismail A., Weyer J. (2022). Coding-complete genome sequences for two confirmed monkeypox cases in South Africa 2022. Microbiol. Resour. Announc..

[22] Djuicy D.D., Sadeuh-Mba S.A., Bilounga C.N., Yonga M.G., Tchatchueng-Mbougua J.B., Essima G.D., Esso L., Nguidjol I.M.E., Metomb S.F., Chebo C. (2024). Concurrent Clade I and Clade II monkeypox virus circulation, Cameroon, 1979–2022. Emerg. Infect. Dis..

[23] Ekpunobi N., Akinsuyi O., Ariri T., Ogunmola T. (2023). The reemergence of monkeypox in Nigeria. Challenges.

[24] Kinganda-Lusamaki E., Amuri-Aziza A., Fernandez-Nuñez N., Makangara-Cigolo J.-C., Pratt C., Vakaniaki E.H., Hoff N.A., Luakanda-Ndelemo G., Akil-Bandali P., Nundu S.S. (2025). Clade I mpox virus genomic diversity in the Democratic Republic of the Congo, 2018–2024: Predominance of zoonotic transmission. Cell.

[25] Cock P.J.A., Antao T., Chang J.T., Chapman B.A., Cox C.J., Dalke A., Friedberg I., Hamelryck T., Kauff F., Wilczynski B. (2009). Biopython: Freely Available Python Tools for Computational Molecular Biology and Bioinformatics. Bioinformatics.

[26] Katoh K., Misawa K., Kuma K., Miyata T. (2002). MAFFT: A Novel Method for Rapid Multiple Sequence Alignment Based on Fast Fourier Transform. Nucleic Acids Res..

[27] Capella-Gutiérrez S., Silla-Martínez J.M., Gabaldón T. (2009). trimAl: A Tool for Automated Alignment Trimming in Large-Scale Phylogenetic Analyses. Bioinformatics.

[28] Larsson A. (2014). AliView: A Fast and Lightweight Alignment Viewer and Editor for Large Datasets. Bioinformatics.

[29] Minh B.Q., Schmidt H.A., Chernomor O., Schrempf D., Woodhams M.D., von Haeseler A., Lanfear R. (2020). IQ-TREE 2: New Models and Efficient Methods for Phylogenetic Inference in the Genomic Era. Mol. Biol. Evol..

[30] Kalyaanamoorthy S., Minh B.Q., Wong T.K.F., von Haeseler A., Jermiin L.S. (2017). ModelFinder: Fast model selection for accurate phylogenetic estimates. Nat. Methods.

[31] Hoang D.T., Chernomor O., von Haeseler A., Minh B.Q., Vinh L.S. (2018). UFBoot2: Improving the Ultrafast Bootstrap Approximation. Mol. Biol. Evol..

[32] Letunic I., Bork P. (2024). Interactive Tree of Life (iTOL) v6: Recent Updates to the Phylogenetic Tree Display and Annotation Tool. Nucleic Acids Res..

[33] Li H. (2013). Aligning sequence reads, clone sequences and assembly contigs with BWA-MEM. arXiv.

[34] Danecek P., Bonfield J.K., Liddle J., Marshall J., Ohan V., Pollard M.O., Whitwham A., Keane T., McCarthy S.A., Davies R.M. (2021). Twelve years of SAMtools and BCFtools. GigaScience.

[35] Cingolani P., Platts A., Wang L.L., Coon M., Nguyen T., Wang L., Land S.J., Lu X., Ruden D.M. (2012). A program for annotating and predicting the effects of single nucleotide polymorphisms, SnpEff: SNPs in the genome of Drosophila melanogaster strain w1118; iso-2; iso-3. Fly.

[36] Adzhubei I.A., Schmidt S., Peshkin L., Ramensky V.E., Gerasimova A., Bork P., Kondrashov A.S., Sunyaev S.R. (2010). A method and server for predicting damaging missense mutations. Nat. Methods.

[37] Jumper J., Evans R., Pritzel A., Green T., Figurnov M., Ronneberger O., Tunyasuvunakool K., Bates R., Žídek A., Potapenko A. (2021). Highly accurate protein structure prediction with AlphaFold. Nature.

[38] Laskowski R.A., MacArthur M.W., Moss D.S., Thornton J.M. (1993). PROCHECK: A program to check the stereochemical quality of protein structures. J. Appl. Crystallogr..

[39] Rodrigues C.H., Pires D.E., Ascher D.B. (2018). DynaMut: Predicting the impact of mutations on protein conformation, flexibility and stability. Nucleic Acids Res..

[40] Ashkenazy H., Abadi S., Martz E., Chay O., Mayrose I., Pupko T., Ben-Tal N. (2016). ConSurf 2016: An improved methodology to estimate and visualize evolutionary conservation in macromolecules. Nucleic Acids Res..

[41] Chakraborty C., Bhattacharya M., Das A., Abdelhameed A.S. (2025). Phylogenetic analyses of the spread of Clade I MPOX in African and non-African nations. Virus Genes.

[42] Langat S.K., Gathii K., Limbaso K., Roba A., Ndia M., Mutai B., Pilarowski G., Ochieng M., Juma B., Onyango C. (2025). Complete genome of an mpox Clade 1b virus from Kenya. Microbiol. Resour. Announc..

[43] Nzoyikorera N., Nduwimana C., Schuele L., Nieuwenhuijse D.F., Koopmans M., Otani S., Aarestrup F.M., Ihorimbere T., Niyomwungere D., Ndihokubwayo A. (2024). Monkeypox Clade Ib virus introduction into Burundi: First findings, July to mid-August 2024. Euro Surveill..

[44] Bbosa N., Nabirye S.E., Namagembe H.S., Kiiza R., Ssekagiri A., Munyagwa M., Bwambale A., Bagonza S., Bosa H.K., Downing R. (2025). Case reports of human monkeypox virus infections, Uganda, 2024. Emerg. Infect. Dis..

[45] Stavrou S., Ross S.R. (2015). APOBEC3 Proteins in Viral Immunity. J. Immunol..

[46] O’Toole Á., Neher R.A., Ndodo N., Borges V., Gannon B., Gomes J.P., Groves N., King D.J., Maloney D., Lemey P. (2023). APOBEC3 deaminase editing in mpox virus as evidence for sustained human transmission since at least 2016. Science.

[47] Martinez T., Shapiro M., Bhaduri-McIntosh S., MacCarthy T. (2019). Evolutionary effects of the AID/APOBEC family of mutagenic enzymes on human gamma-herpesviruses. Virus Evol..

[48] Abdi B., Lambert-Niclot S., Wirden M., Jary A., Teyssou E., Sayon S., Palich R., Tubiana R., Simon A., Valantin M.-A. (2021). Presence of HIV-1 G-to-A mutations linked to APOBEC editing is more prevalent in non-B HIV-1 subtypes and is associated with lower HIV-1 reservoir. J. Antimicrob. Chemother..

[49] Liu Y. (2020). A code within the genetic code: Codon usage regulates co-translational protein folding. Cell Commun. Signal..

[50] Sauna Z.E., Kimchi-Sarfaty C. (2011). Understanding the contribution of synonymous mutations to human disease. Nat. Rev. Genet..

[51] Karumathil S., Raveendran N.T., Ganesh D., Kumar NS S., Nair R.R., Dirisala V.R. (2018). Evolution of Synonymous Codon Usage Bias in West African and Central African Strains of Monkeypox Virus. Evol. Bioinform..

[52] Zhu J., Yu J., Qin H., Chen X., Wu C., Hong X., Zhang Y., Zhang Z. (2023). Exploring the key genomic variation in monkeypox virus during the 2022 outbreak. BMC Genom. Data.

[53] Shan K.-J., Wu C., Tang X., Lu R., Hu Y., Tan W., Lu J. (2024). Molecular Evolution of Protein Sequences and Codon Usage in Monkeypox Viruses. Genom. Proteom. Bioinform..

[54] Zhang S., Wang F., Peng Y., Gong X., Fan G., Lin Y., Yang L., Shen L., Niu S., Liu J. (2024). Evolutionary trajectory and characteristics of Mpox virus in 2023 based on a large-scale genomic surveillance in Shenzhen, China. Nat. Commun..

[55] Zandi M., Shafaati M., Hosseini F. (2023). Mechanisms of immune evasion of monkeypox virus. Front. Microbiol..

[56] Boyle K.A., Arps L., Traktman P. (2007). Biochemical and genetic analysis of the vaccinia virus D5 protein: Multimerization-dependent ATPase activity is required to support viral DNA replication. J. Virol..

[57] Bloom J.D., Labthavikul S.T., Otey C.R., Arnold F.H. (2006). Protein stability promotes evolvability. Proc. Natl. Acad. Sci. USA.

[58] Chen H., Xu Y., Li Y., Qu H.-Q., Hakonarson H., Li J., Xia Q. (2025). Genomic variation and impact on the proteins of Mpox virus. J. Infect..

[59] Tokuriki N., Tawfik D.S. (2009). Stability effects of mutations and protein evolvability. Curr. Opin. Struct. Biol..

