# Supplementary material for: Phylogenetic Analysis of the Mpox Virus in Sub-Saharan Africa (2022–2024)
Source: Biology (Basel). 2025 Jun 26;14(7):773. doi: 10.3390/biology14070773 (PMC12292466; doi:10.3390/biology14070773)
Supplement: Supplementary file 1 [file biology-14-00773-s001.zip › supplimentary_files/supplimentary_files/File_S6_Functional_Analysis.zip!/File_S6_Functional_Analysis/fold_opg_145_gp_124/consurf_colored_seq.pdf]

ConSurf Results for job:opg 145gp124 date:27/05/2025

```
1           11          21          31          41
MSLLKMEYNL  YAELKKMTCG  QTISLFNEDG  DFVEVEPGSS  FKFLIPKGFY
eeebebeeeb  beebeebeee  ebbbbbeeee  ebbebbeebe  bebbbbbbbb
 f    f  s                  ss         fs   s        s s  

51          61          71          81          91
SSPCVKTSLV  FETLTTTDNK  ITSINPTNAP  KLYPLQRKVV  SEVVSNMRKM
beeebebeee  eeebeeeeee  beeeeeeebe  ebbebeeebb  eebbeebeee
                    f            f   ss  f  s             

101         111         121         131         141
IELKRPLYIT  LHLACGFGKT  ITTCYLMTTH  GRKTIICVPN  KMLIHQWKTQ
eeeeeebbbb  bbbbbebeee  bbbbbbbeee  eeebbbbbee  ebbbeebeeb
    f  s s  s s sfsfff       s   f    f     ff  f    f    

151         161         171         181         191
VEAVGLEHKI  SIDGVSSLLK  ELKTQSPDVL  IVVSRHLTND  AFCKYINKHY
beebebebbb  bbeebeebbe  ebeeeebebb  bbbbeebeee  ebbeebeeeb
            s  f   s             s   s  ff  f    ss  s    

201         211         221         231         241
DLFILDESHT  YNLMNNTAVT  RFLAYYPPMM  CYFLTATPRP  ANRIYCNSII
ebbbbbebbb  bbbbeeebbb  ebbbeeeeeb  bbbbebeeee  beebbbeebb
f    sfss   ssssf         s   ff    ssssfsfff    f    f   

251         261         271         281         291
NIAKLSDLKK  TIYIVDSFFE  PYSTDNIRNM  VKRLDGPSNK  YHIYTEKLLS
ebbebeebee  bbebbeebbe  eebeeebeeb  beebeeeeee  eebbbeebbb
f  f f   f                                  ff  ff  sff s 

301         311         321         331         341
VDEPRNQLIL  DTLVEEFKSG  TINRILVITK  LREHMVFFYK  RLLDLFGAEV
eeeeeeebbb  eebbeebeee  ebeebbbbbe  beebbbbbbe  ebbeebeeeb
ff  ff  s    f             f     f  sf ss                 

351         361         371         381         391
VFIGDAQNRR  TPDMVKSIKE  LNRFIFVSTL  FYSGTGLDIP  SLDSLFICSA
bbbbebeeee  eeebbeebee  eeebbbbbbb  bbebebbebe  ebebbbbbbb
   sf                      s   ss    s sfs f     sf s     

401         411         421         431         441
VINNMQIEQL  LGRVCRETEL  LDRTVYVFPN  TSIKEIKYMI  GNFVQRIISL
bbeebebeeb  beebbeeeee  eeebbbbbbe  bbbeebeebb  bbbbeebbbb
s f    ff    ff sf        f    s    ss   s          ff    

451         461         471         481         491
SVDKLGFKQE  SYQKHQESEP  ASVPTSSREE  RVLNRIFNSQ  NR
bbeebebeee  eeeeeeeeee  eebeeeeeee  ebbebbbeee  ee
    sfs                  f                 f f   f
```

The conservation scale:

1 2 3 4 5 6 7 8 9

Variable — Average — Conserved

e - An exposed residue according to the neural network algorithm.
b - A buried residue according to the neural network algorithm.
f - A predicted functional residue (highly conserved and exposed).

s - A predicted structural residue (highly conserved and buried).
x - Insufficient data - the calculation for this site was
performed on less than 10% of the sequences.
