# Supplementary material for: Phylogenetic Analysis of the Mpox Virus in Sub-Saharan Africa (2022–2024)
Source: Biology (Basel). 2025 Jun 26;14(7):773. doi: 10.3390/biology14070773 (PMC12292466; doi:10.3390/biology14070773)
Supplement: Supplementary file 1 [file biology-14-00773-s001.zip › supplimentary_files/supplimentary_files/File_S6_Functional_Analysis.zip!/File_S6_Functional_Analysis/fold_opg_210_gp_173/consurf_colored_seq.pdf]

ConSurf Results for job:OPG 210-gp 173 date:27/05/2025

```
1           11          21          31          41
MNFQKLSLAI  YLTVTCSWCY  ETCMRKTALY  HDIQLEHVED  NKDSVASLPY
eebeebbbbb  bbbbbbbbeb  eebbeeebbe  eeeebeeeee  eeeebbebee
                           sff   f  ff       f           f

51          61          71          81          91
KYLQVVKQRE  RSRLLATFNW  TDIAEGVRNE  FIKICDINGT  YLYNYTIAVS
ebbeebeeee  ebebbbebeb  eebeeebeee  bbeebeeeeb  ebbbbbbebb
f        f    f           s                f    f  s ss

101         111         121         131         141
IIIDSTEELP  TVTPITTYEP  SIYNYTIDYS  TVITTEELQV  TPTYAPVTTP
bbbebbeeee  eeeebeeeee  ebbbbbbebb  bbbeeeebeb  eeeeeeeeee
                 f                                   f

151         161         171         181         191
LPTSAVPYDQ  RSNNNVSTIS  IQVLSKILGV  NETELTNYLI  MHKNDTVDNN
eeeeeeeeee  eeeeebbebb  bbbbbebbeb  eebebbbbbb  eeeeeeeeee
       f                        f   f                   f

201         211         221         231         241
TMVDDETSDN  NTLHGNIGFL  EINNCYNVSV  SDASFRITLV  NDTSEEILLM
eeeeeeeeee  eebebbbbbb  ebeebbebbb  eebbbebbbe  eeebeebbbb
                              f s                   f

251         261         271         281         291
LTGTSSSDTF  ISSTNITECL  KTLINNVSIN  DVLITQNMNV  TSNCDKCSMN
bbeeeeeebb  bebeebbebb  eebbeeebee  ebebbbebeb  eeebeebbbe
    f         s              f                   f     s

301         311         321         331         341
LMASVIPAVN  EFNNTLMKIG  VKDDENNTVY  KYYNCKLTTN  STCDELINLD
bbbbbbebbe  ebeeebeebb  beeeeeeeee  ebbebeeeee  eebeebbebe
 s  s         f f   s    f    f           f         f

351         361         371         381         391
EVINNITLTN  IIHNSVSTTN  SRKRRDLNDE  FEFSTSKELD  CLYESYGVND
ebbbbbbbbb  bbeeebeeee  eeeeeebeee  beeeeeeebe  bebeeeeeee
  s   s  s               f  f              f

401         411         421         431         441
DISHCFASPR  RRRSDDKKEY  MDMKLFDHAK  KDLGIDSVIP  RGTTHFQVGA
ebeebbeeee  eeeeeeeeeb  eebebbeebe  eeeeeeebbe  ebeeebebbb
         f    fff               s            f     ff f

451         461         471         481         491
SGASGGVVGD  SFPFQNVKSR  ASLLAEKIMP  RVPITATEAD  LYATVNRQPK
eeeeebbbbe  eebbeebeee  beebbeebbe  ebeeeeeeee  bbbebeeeee
      s  f         f        s f  f           f           f

501         511         521         531         541
LPAGVKSTPF  TEALVSTINQ  KLSNVREVTY  ASLNLPGSSG  YVHRPSDSVI
eeeeeeeeeb  eebbbbebee  ebeebeebbb  bebebeeeee  eeeeeeeebe
 f           f    f            s

551         561         571         581         591
YSSIRRSRLP  SDSDSDYEDI  QTVVKEYNER  YGRSVSRTQS  SSSESDFEDI
bbebeeeeee  eeeeeebeeb  eebbeebeee  eeeeeeebee  eeeeeebeeb
              f ff ff     s                      f  ffsf
```

```
601        611        621        631        641
DTVVREYRQK YGNAMAKGRS SSPKPDPLYS TVKKTTKSLS TGVDIVTKQS
eebbeebeee eeeeeeeeee eeeeeeeeee ebeeeeeeeb ebbebbeeee
     f     f        f      f   f    f        s

651        661        671        681        691
DYSLLPDVNT GSSIVSPLTR KGATRRRPRR PTNDGLQSPN PPLRNPLPQH
eeebbeebee eebbbbebee eebbeeeeee eeeeeeeeee eeeeeeeeee
         f       f ff     f

701        711        721        731        741
DDYSPPQVHR PPPLPPKPVQ NPPQLPPRPV GQLLPPPIDQ PDKGFSKFVS
eeeeeeeeee eeeeeeeeee eeeeeeeeee eeebeeeeee eeeebbeeeb
               f           ff

751        761        771        781        791
PRRCRRASSG VICGMIQSKP NDDTYSLLQR SKIEPEYVEV GNGIPKNNVP
eeebeeeeee bbbbbbeeee eeeebbbeee eeeeeeeeeb eeebeeeebe
 ffsff  ff s s   f  f      s           f f    f     s

801        811        821        831        841
VIGNKHSKKY TSTMSKISTK FDKSTAFGAA MLLTGQQAIS QQTRSTTLSR
bbeeeeeeee eeebeebbee beebbbbbbb bbbbbeebbe eebeeebbee
         f             ffs     s s    f s    f f

851        861        871        881        891
KDQMSKEEKI FEAVTMSLST IGSTLTSAGM TGGPKLMIAG MAITAITGII
eeeeeeeeeb bebbbbbbbb bbbebbbbbb bbbbebbbbb bbbbbbbbbb
 f f   f    f s   ss      s   s     s     s      s s s

901        911        921        931        941
DTIKDIYYMF SGQERPVDPV IKLFNKYTGL MSDNNKMGVR KCLTPGDDTL
ebbeebbbbb eeeeeeeeeb bebbbbbbbe beeeeeeebe ebebeeeebb
f   f         f f ff   f     s         f f f  sf f

951        961        971        981        991
IYIAYRNDTS FKQNTDAMAL YFLDVIDSEI LYLNTSNLVL EYQLKVACPI
bbbbbeeeee eeeeeeebbb bbbebbebeb bbbebbebbb ebebebbbeb
    s f    ff          ssf s s    s fssf    f  s s sfs

1001       1011       1021       1031       1041
GTLRSVDVDI TAYTILYDTA DNIKKYKFIR MATLLSKHPV IRLTCGLAAT
bbbebeebeb bbbbbbbeee eebeebebbb bbbbbeeeeb bebbbeeebe
s sfs f fs   s   s         s         sf ff    sssf

1051       1061       1071       1081       1091
LVIKPYEVPI SDMQLLKMAT PGEPESTKSI PSDVCDRYPL KKFYLLAGGC
bbbeebebbb eebebbeebe eeeeeeeeee eeebbeeeeb eebbbbbebb
s   fsf    f sfsfff f ffff  f ff fff sf  f  f sss   ss

1101       1111       1121       1131       1141
PYDTSQTFIV HTTCSILLRT ATRDQFRNRW VLQNPFRQEG TYKQLFTFSK
eeeeeebbbb bbbbbbbbee beeeebeeeb bbeeebeeee ebeebbebbe
fff ff  ss  sss s      f f   ffs  sfffs fff   ffssfs f

1151       1161       1171       1181       1191
YDFNDTIIDP NGVVGHASFC TNRSSNQCFW SEPMILEDVS SCSSRTRKIY
eebeebbbee eeeeeebebb eeeeeeebbb bebbbbeebe ebeeebeebb
 fsf s   f f    f   s  f  f  s s  f s  f     s  f ff
```

```
1201        1211        1221        1231        1241
VKLGIFNAEG  FNSFVLNCPT  GSTPTYIKHK  NADSNNVIIE  LPVGDYGTAK
bebebbebeb  bebbbbebee  eeeeeebeee  eeeeeebbbe  bbbeeebebe
 f   s   s  s s    sff  ffff                 f      ff   f

1251        1261        1271        1281        1291
LYSATKPSRI  AVFCTHNYDK  RFKSDIIVLM  FNKNSGIPFW  SMYTGSVTSK
bbbeeeeeeb  bbbbbbebee  ebebebbbbb  bbebeebebb  bbeeeeeeee
  s          s s  f f   f fsf

1301        1311        1321        1331        1341
NRMFATLARG  MPFRSTYCDN  RRRSGCYYAG  IPFHEDSVET  DIHYGPEIML
eebbbebbee  bbbebebbee  eeeeebebbe  bebeeeebeb  ebeebbebbb
               fs  sff   ff  s                         s

1351        1361        1371        1381        1391
KETYDINSID  PRVITKSKTH  FPAPLSVKFM  VDNLGNGYDN  PNSFWEDAKT
eeeeeeeebe  eebbeebeee  beeebebebe  beebeeeeee  eeebbeebee
 fff   f f       f f    sf             sf  f       ss fs

1401        1411        1421        1431        1441
KKRTYSAMTI  KVLPCTVRNK  NIDFGYNYGD  IISNMVYLQS  TSQDYGDGTK
eeeebbbbbb  ebbebebeee  ebebbbbbbb  bbbbbbbbee  eeeeeeeeee
 fffss          sf ff                 s   s ff      ff

1451        1461        1471        1481        1491
YTFKSVTRSD  HECESSLDLT  SKEVTVTCPA  FSIPRNISTY  EGLCFSVTTS
eebeeeeeee  eebebbbebe  eeebebebee  bebeeebeeb  ebbbbbbbeb
f s           s   s          s s     fs f       fs s    fs

1501        1511        1521        1531        1541
KDHCATGIGW  LKSSGYGKED  ADKPRACFHH  WNYYTLSLDY  YCSYEDIWRS
eeebeeeeee  beeeeeeeee  eeeeebbeee  bbeebebbeb  bbbbeeebee
 ffsff       f  ff          f s             f   ss

1551        1561        1571        1581        1591
TWPDYDPCKS  YIHIEYRDTW  IESNVLQQPP  YTFEFIHDNS  NEYVDKEISN
eeeeeeebeb  ebbbebeebb  bebeeeeeee  eebebeeeee  eebbeeebbe
  f ff s s  f   fs   s   f  fff ff  ffsfs    f  ffs  f

1601        1611        1621        1631        1641
KLNDLYNEYK  KIMEYSDGSL  PASINRLAKA  LTSEGREIAS  VNIDGNLLDI
ebeebeeebe  ebbebeeeeb  eebbeebbeb  beeebeebbb  bbbebebbbb
    sf  s      f ffffs  f ssffss    sf f f s    ss fsf s

1651        1661        1671        1681        1691
AYQADKEKMA  DIQTRINDII  RDLFIHTLSD  KDIKDIIESE  EGKRCCIIDV
beeeeeeebb  ebeeebeebb  eebbbbbbee  eebeebbeee  eeeebbbbeb
sffff ff      f  s            ssf    f   s f f     fss  f

1701        1711        1721        1731        1741
KNNLVKKYYS  IDNYLCDTLD  DYIYTSVEYN  KSYVLVNDTY  MSYDYLESSG
eeeebeeebe  beebbbbebe  eebbbeeeee  eebbbbebbb  bbbebbeebe
 f    f s    f s s   f  ff           fs   f          s  s

1751        1761        1771        1781        1791
VVVLSCYEMT  IISLDTKDAK  DAIEDVIVAS  AVAEALNDMF  KEFDKNVSAI
bbbbbbbebb  bbbbeeeebe  eebeeebbeb  bbbebbeebb  eebeeebbbb
     s       s s f         f  s     s     f      f f f s
```

```
1801       1811       1821       1831       1841
IIKEEDNYLN SSPDIYHIIY IIGGTILLLL VIILILAIYI ARNKYRTRKY
bbeeeeeebe eeeebbbbbb bbbbbbbbbb bbbbbbbbbb bbeeeeeeeb
    f  f                             s               s

1851       1861       1871
EIMKYDNMSI KSEHHDSLET VSMEIIDNRY
ebbeeeeeee eeeeeebbeb bbbebbeeee
                       ss
```

The conservation scale:

1 2 3 4 5 6 7 8 9

Variable Average Conserved

e - An exposed residue according to the neural network algorithm.
b - A buried residue according to the neural network algorithm.
f - A predicted functional residue (highly conserved and exposed).
s - A predicted structural residue (highly conserved and buried).
x - Insufficient data - the calculation for this site was performed on less than 10% of the sequences.
