# Supplementary material for: Phylogenetic Analysis of the Mpox Virus in Sub-Saharan Africa (2022–2024)
Source: Biology (Basel). 2025 Jun 26;14(7):773. doi: 10.3390/biology14070773 (PMC12292466; doi:10.3390/biology14070773)
Supplement: Supplementary file 1 [file biology-14-00773-s001.zip › supplimentary_files/supplimentary_files/File_S7_Ramachandran_plots.zip!/File_S7_Ramachandran_plots/File_S7_Ramachandran_plots/OPG_145_Ramachandran Plot.pdf]

PROCHECK

Ramachandran Plot

saves

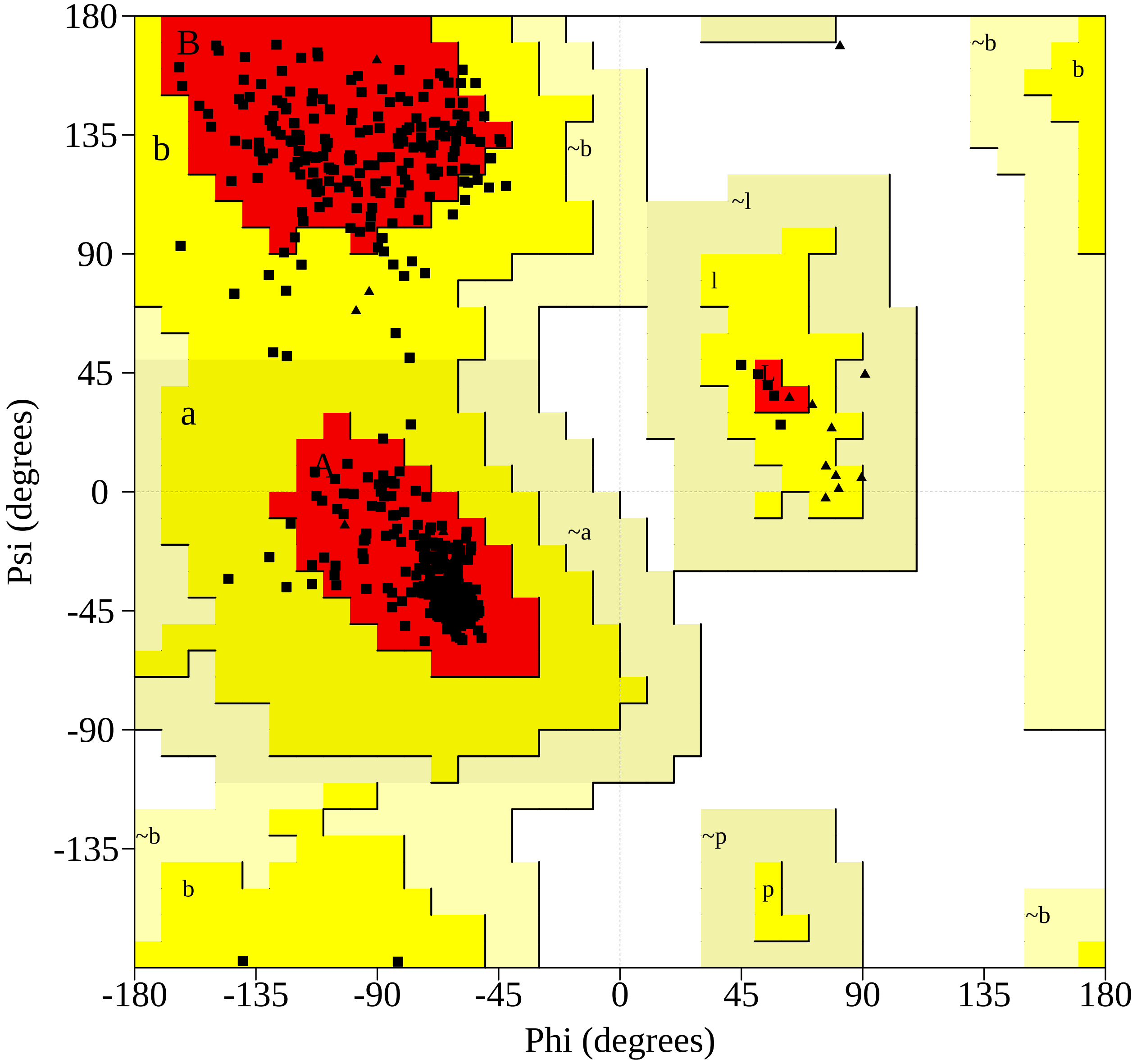

Plot statistics

Residues in most favoured regions [A,B,L]	415	92.0%
Residues in additional allowed regions [a,b,l,p]	36	8.0%
Residues in generously allowed regions [~a,~b,~l,~p]	0	0.0%
Residues in disallowed regions	0	0.0%
	----	------
Number of non-glycine and non-proline residues	451	100.0%
Number of end-residues (excl. Gly and Pro)	2	
Number of glycine residues (shown as triangles)	18	
Number of proline residues	21	

Total number of residues	492	

Based on an analysis of 118 structures of resolution of at least 2.0 Angstroms and R-factor no greater than 20%, a good quality model would be expected to have over 90% in the most favoured regions.

saves_01.ps
