# Supplementary material for: Phylogenetic Analysis of the Mpox Virus in Sub-Saharan Africa (2022–2024)
Source: Biology (Basel). 2025 Jun 26;14(7):773. doi: 10.3390/biology14070773 (PMC12292466; doi:10.3390/biology14070773)
Supplement: Supplementary file 1 [file biology-14-00773-s001.zip › supplimentary_files/supplimentary_files/File_S7_Ramachandran_plots.zip!/File_S7_Ramachandran_plots/File_S7_Ramachandran_plots/OPG_210_Ramachandran Plot.pdf]

PROCHECK

Ramachandran Plot

saves

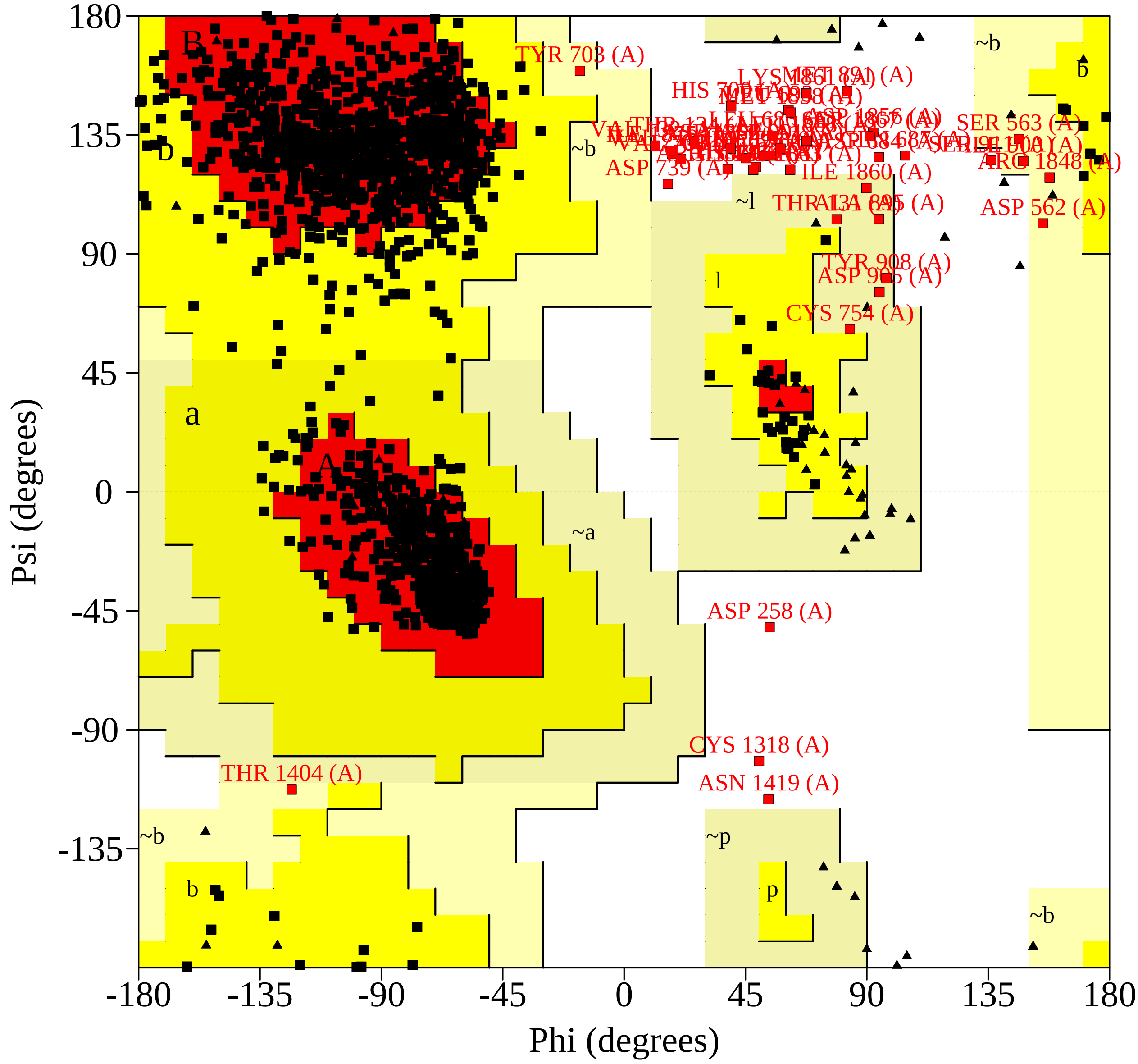

Plot statistics

Residues in most favoured regions [A,B,L]	1487	87.3%
Residues in additional allowed regions [a,b,l,p]	175	10.3%
Residues in generously allowed regions [~a,~b,~l,~p]	12	0.7%
Residues in disallowed regions	30	1.8%
	----	------
Number of non-glycine and non-proline residues	1704	100.0%
Number of end-residues (excl. Gly and Pro)	2	
Number of glycine residues (shown as triangles)	81	
Number of proline residues	93	

Total number of residues	1880	

Based on an analysis of 118 structures of resolution of at least 2.0 Angstroms and R-factor no greater than 20%, a good quality model would be expected to have over 90% in the most favoured regions.
